# Investigation of damage evolution in asphalt concrete under coupled ultraviolet aging and freeze-thaw cycles

**DOI:** 10.1038/s41598-026-58169-8

**Published:** 2026-06-16

**Authors:** Zhengyi Yu, Yirui Cao, Xiaoyu Xu, Lijun Sun, Ziliang Ma, Jin Yang, Wu Bo, Qiao Dong, Zhen Liu

**Affiliations:** 1 School of Urban Construction, Hangzhou Polytechnic, Fuyang, Hangzhou, 311402 Zhejiang Province People’s Republic of China; 2 School of Transportation, Southeast University, Jiangning District, Nanjing, 211189 Jiangsu China; 3 CCCC Second Highway Consultants Co., Ltd., Wuhan, 430056 Hubei Province China; 4 School of Engineering, Plateau Major Infrastructure Smart Construction and Resilience Safety Technology Innovation Center, Tibet University, Lhasa, 710065 Tibet Autonomous Region China; 5 Institute of Space and Earth Information Science, Fok Ying Tung Remote Sensing Science Building, The Chinese University of Hong Kong, Hong Kong SAR, China

**Keywords:** Asphalt concrete, UV aging, Freeze-thaw cycles, Coupled damage, Semi-circular bending test, Engineering, Materials science

## Abstract

To clarify the degradation law of crack resistance of asphalt concrete under the coupled action of UV aging and freeze–thaw cycles, semi-circular bending tests were conducted on unaged and UV-aged specimens subjected to varying numbers of freeze–thaw cycles. Key parameters, including air void content, flexural tensile strength, flexural stiffness, failure displacement, and fracture energy, were measured, and their effectiveness in characterizing coupled damage was evaluated using a damage index and correlation analysis. The results indicate that increasing freeze–thaw cycles lead to a gradual rise in air void content, accompanied by continuous reductions in flexural tensile strength, pre-peak fracture energy, and total fracture energy, demonstrating a significant deterioration in internal compactness and crack resistance. Under identical freeze–thaw conditions, specimens exposed to combined UV aging and freeze–thaw effects exhibit higher air void content and further reductions in strength and fracture energy compared to those subjected to freeze–thaw cycles alone, indicating that UV aging exacerbates freeze–thaw damage. Although flexural stiffness shows an overall decreasing trend, coupled-damage specimens maintain relatively higher values than unaged specimens, reflecting the stiffening effect induced by UV aging. Damage index analysis identifies flexural tensile strength as the most sensitive indicator of both freeze–thaw and coupled damage, followed by fracture energy and air void content. Correlation analysis reveals a general negative relationship between air void content and major crack-resistance indices, while flexural tensile strength shows a strong positive correlation with pre-peak fracture energy. Overall, flexural tensile strength and pre-peak fracture energy are recommended as key indicators for evaluating the evolution of crack resistance under coupled environmental effects.

## Introduction

Asphalt pavements in high-altitude, severely cold regions with large temperature differences are subjected for long periods to intense ultraviolet (UV) radiation, frequent freeze-thaw action, and repeated moisture migration, and therefore suffer particularly severe surface cracking, raveling, and durability degradation^1^. Existing studies have shown that environmental damage in asphalt concrete in such regions is not dominated by a single factor, but rather results from the coupled and progressive interaction among surface aging, interfacial deterioration, and internal structural degradation^2,3^. Analyses of the natural environment along the Qinghai-Tibet Highway indicate that freeze-thaw action in cold regions is characterized by high frequency, large temperature differentials, and pronounced alternation between severe and mild cycles, and that its damage intensity depends not only on cycle number but also on freezing temperature, thawing temperature, and the temperature history^4^. Meanwhile, high-altitude areas are generally subjected to strong UV radiation, and the pavement surface undergoes significant photo-oxidative aging at an early service stage^5^. Therefore, discussing damage evolution in asphalt pavements in these regions from the perspective of freeze-thaw cycles or UV aging alone can no longer realistically reflect the in-service deterioration process of the material.

The damage characteristics of asphalt concrete under freeze-thaw cycles have been systematically investigated. The literature generally agrees that freeze-thaw damage is initiated by phase transformation of pore water^6^. At low temperature, water entering pores and interfaces freezes and expands, causing volume change and redistribution of internal stresses. During thawing, moisture continues to migrate and repeated hydraulic disturbance further weakens asphalt-aggregate adhesion and the cohesion of asphalt mastic, promoting the initiation, propagation, and accumulation of existing micropores and microcracks^7^. Based on uniaxial compression tests, Li Ping et al., showed that the mechanical properties of continuously dense-graded asphalt mixture continuously decrease as the number of freeze-thaw cycles increases, while the freeze-thaw damage variable gradually increases and can be well described by an exponential function^6^. Their constitutive damage model can satisfactorily capture the stress-strain response of the material under freeze-thaw action. Tan Yiqiu et al., from the perspective of durability damage, proposed a general damage model and a corresponding life prediction method for asphalt mixtures under freeze-thaw conditions, demonstrating that freeze-thaw action manifests not only as performance loss but also as cumulative damage evolution^8^. Li Jin et al., further reported that, among several mechanical indices, low-temperature fracture energy is the most sensitive to freeze-thaw cycles and can more promptly reflect material deterioration, and that the damage evolution model established based on this index shows high fitting accuracy^9^. Wu Jinrong et al., also found that freeze-thaw cycles significantly reduce the fatigue life of asphalt concrete, with more severe deterioration at low temperature and in corrosive media^10^. These studies indicate that the essence of freeze-thaw cycles lies in the progressive internal structural damage induced by pore-water phase transformation and interface weakening, and that the main consequences are a reduction in material compactness, deterioration of crack resistance, and shortening of fatigue life.

Unlike freeze-thaw cycles, which mainly act on the internal water-bearing structure, UV aging first occurs at the surface of asphalt concrete and constitutes an important precursor damage during service. Existing reviews generally indicate that UV radiation has sufficiently high photon energy to induce bond scission, free-radical generation, oxidative polycondensation, and colloidal structure reorganization in asphalt molecules, leading to a decrease in light fractions and an increase in heavy fractions^11,12^, which in turn manifests as hardening, embrittlement, and reduced adhesion^13^. Zeng et al., further showed that UV aging has a pronounced gradient characteristic: direct photo-aging is confined to an extremely thin surface layer, but the aging products formed at the surface can further diffuse inward and cause secondary aging of internal asphalt^14^. In a study on warm-mix asphalt mixtures, Yu Yanzhong et al., reported that, with increasing UV exposure duration, nanoscopic adhesion continuously decreased, while the freeze-thaw splitting strength ratio and retained stability ratio gradually declined and the freeze-fracture temperature gradually increased; moreover, nanoscopic adhesion exhibited good linear correlations with these macroscopic indices^15^. This indicates that UV aging first weakens the asphalt film and the asphalt-aggregate interface, and is then further translated into reduced moisture resistance and low-temperature crack resistance at the mixture scale. In other words, although UV aging mainly initiates at the surface, the interfacial embrittlement and adhesion deterioration it causes create less favorable conditions for moisture ingress and subsequent freeze-thaw damage propagation^14^.

Based on the above single-factor damage mechanisms, some recent studies have begun to focus on the coupling between UV aging and freeze-thaw cycles and have confirmed a significant synergistic effect between them. He et al., pointed out, under intense UV radiation and large temperature fluctuations, that at the binder scale UV radiation exerts a more pronounced influence on penetration, ductility, and chemical composition, whereas at the mixture scale freeze-thaw action acts as the direct damage factor and UV aging amplifies freeze-thaw deterioration by accelerating surface hardening and interfacial embrittlement^16^. Xie et al., established an environmental cycle consisting of freezing, UV irradiation, and thawing for hot recycled asphalt mixtures, and found that, with increasing environmental exposure, high-temperature stability, low-temperature performance, and moisture stability all continuously deteriorated, among which the low-temperature crack-resistance and moisture-stability indices were more sensitive^17^. Based on the FAM system, Li et al., further showed that UV exposure, freeze-thaw cycles, and their coupled action all reduce fatigue life and self-healing capacity, with the most pronounced deterioration occurring under coupled conditions^18^. Cheng et al., also reported for porous asphalt pavements that, under the same number of freeze-thaw cycles, the raveling loss rate increases with increasing UV-aging times; under the same UV exposure history, increasing freeze-thaw cycles likewise continuously aggravate surface raveling^19^. These studies collectively demonstrate that the effects of UV aging and freeze-thaw cycles on asphalt concrete are not simply additive, but rather form a continuous damage chain through surface aging, interface weakening, moisture ingress, and freeze-thaw damage propagation.

Fracture-based testing has been increasingly used to evaluate environmental damage in asphalt mixtures because it can directly characterize crack initiation and propagation rather than only residual strength. Among available fracture tests, the notched semi-circular bending (NSCB) test has been widely adopted for asphalt concrete owing to its simple specimen preparation, stable crack path, and ability to derive multiple strength-, deformation-, stiffness-, and energy-based indicators from a single loading process. Previous studies have applied SCB or NSCB tests to evaluate moisture- and freeze–thaw-induced fracture deterioration, showing that freeze–thaw cycles significantly reduce fracture toughness and fracture energy by promoting internal void growth, interface weakening, and microcrack propagation. Recent studies on UV aging–freeze–thaw coupling have further confirmed the accelerated degradation of mixture performance under combined environmental actions. However, most available studies have emphasized the overall deterioration of individual mechanical or fracture indices, while the relative sensitivity and stability of different NSCB-derived indicators under coupled UV aging–freeze–thaw damage remain insufficiently compared. This gap motivates the present study to establish a unified NSCB-based evaluation framework for selecting robust crack-resistance indicators under coupled environmental damage.

Although recent studies have confirmed the synergistic deterioration of asphalt materials under coupled UV aging and freeze–thaw cycles, most existing work has focused on the overall degradation of road performance, moisture stability, fatigue behavior, or raveling resistance. The available studies have demonstrated that UV aging can amplify freeze–thaw-induced deterioration, but the evaluation of crack-resistance degradation is still mainly based on individual mechanical indices. In particular, the relative sensitivity, stability, and interpretability of strength-, stiffness-, displacement-, and energy-based indices under coupled UV aging–freeze–thaw damage have not been systematically compared within a unified fracture-testing framework. This limitation makes it difficult to determine which indicators are most suitable for characterizing coupled damage in asphalt concrete.

The incremental contribution of this study is therefore to establish a notched semi-circular bending (NSCB)-based evaluation framework for asphalt concrete subjected to freeze–thaw damage and coupled UV aging–freeze–thaw damage. Within this framework, air void content, flexural tensile strength, flexural stiffness, failure displacement, pre-peak fracture energy, post-peak fracture energy, and total fracture energy were jointly analyzed from the perspectives of internal structural change, load-bearing capacity, deformation resistance, and fracture energy dissipation. Damage index analysis and Pearson correlation analysis were further used to compare the response sensitivity and stability of these indicators. By identifying flexural tensile strength and pre-peak fracture energy as relatively robust indicators, this study provides a more targeted basis for evaluating crack-resistance deterioration of asphalt concrete in high-altitude cold regions.

## Experimental program and evaluation methods

### Semi-circular bending test

In this study, semi-circular bending (SCB) tests were used to evaluate the crack resistance of asphalt concrete under freeze-thaw cycling and coupled UV aging-freeze-thaw action. The correlations between the crack-resistance indices and the number of freeze-thaw cycles were also analyzed to identify key evaluation indices for subsequent investigation^20^.

**Fig. 1:**
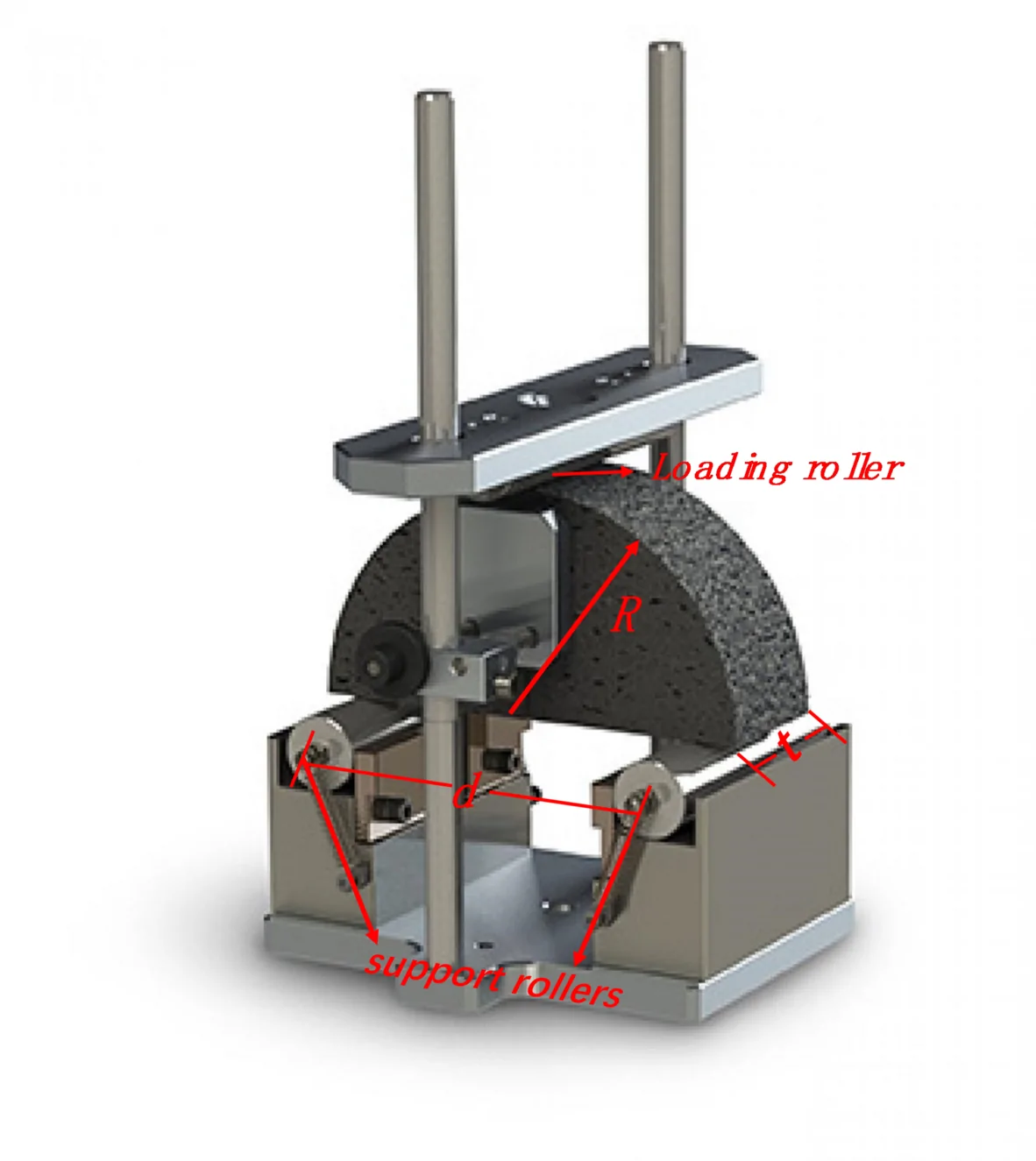
Schematic of the notched semi-circular bending (NSCB) test configuration.

The SCB test adopted a three-point loading configuration. The semi-circular specimen was supported by two cylindrical rollers at the bottom, and a vertical load was applied at the top until specimen fracture. The support span was taken as 0.8 times the specimen diameter. The specimen diameter was 150 mm, corresponding to a support span of 120 mm. The test temperature was 5 °C, the loading rate was 5 mm/min, and the specimen thickness was 50 mm. A schematic of the test setup is shown in Fig. 1.

### Evaluation indices for crack resistance

To comprehensively characterize the crack resistance of asphalt concrete, four commonly used indices in SCB testing were selected from the perspectives of strength, stiffness, deformation capacity, and energy dissipation capacity, namely flexural tensile strength, flexural stiffness, failure displacement, and fracture energy.

Flexural tensile strength *σ*_*m*_ characterizes the ultimate load-bearing capacity of the asphalt concrete specimen during loading and is calculated by Eq. (1)^21^.

1 $$\:\begin{array}{c}{\sigma\:}_{m}=\frac{4.8{F}_{max}}{WD} \end{array}$$

where:

*σ*_*m*_ — maximum tensile stress at the bottom of the specimen, i.e., flexural tensile strength (MPa); *F*_*max*_ — peak load recorded during the test (N); *W* — thickness of the semi-circular specimen (mm); *D* — diameter of the semi-circular specimen (mm).

Flexural stiffness *S* characterizes the ability of the semi-circular specimen to resist deformation and cracking at the initial stage of loading and reflects the crack-resistance stiffness of the material in the elastic stage. It is calculated by Eq. (2)^22^.

2 $$\:\begin{array}{c}S=\frac{0.5\times\:{F}_{max}}{{d}_{t}} \end{array}$$

where:

*S* — flexural stiffness (kN/mm); *d*_*t*_ — displacement corresponding to one-half of the peak load (mm).

Failure displacement *u*_*0*_ originally refers to the vertical actuator displacement at the moment when the specimen fails by fracture. Since it is difficult in actual testing to accurately capture the displacement at the instant of final fracture^23,24^, the displacement corresponding to the peak load was adopted in this study as an engineering approximation.

Fracture energy represents the energy consumed per unit crack-propagation area and was calculated from the area enclosed by the load–displacement curve, as expressed in Eq. (3)^25^.

3 $$\:\begin{array}{c}{G}_{f}=\frac{{W}_{f}}{A} \end{array}$$

where:

*G*_*f*_ — fracture energy (J/mm^2^); *W*_*f*_ — fracture work calculated from the load–displacement curve (J); *A* — ligament area of the notched semi-circular specimen (mm^2^), calculated as (mm^2^).

$$\:A=\left.\left(\frac{D}{2}-{a}_{0}\right.\right)W$$

where *D* is the specimen diameter, *a*_*0*_ is the notch length, and *W* is the specimen thickness. In this study, *D* = 150 mm, *a*_*0*_ = 15 mm, and *W* = 50 mm.

To distinguish the energy characteristics at different fracture stages, three fracture-energy indices were used in this study: pre-peak fracture energy *G*_*af*_, post-peak fracture energy *G*_*bf*_, and total fracture energy *G*_*f*_. The pre-peak fracture energy *G*_*af*_ was calculated from the initial loading point to the peak-load point and mainly reflects the energy required for crack initiation and stable crack propagation. The post-peak fracture energy *G*_*bf*_ was calculated from the peak-load point to the point at which the load decreased to one-half of the peak load, representing the residual energy dissipation after crack formation. The total fracture energy *G*_*f*_ was defined as the sum of the pre-peak and post-peak fracture energies.

Under the present SCB test conditions, the descending branch of the load–displacement curve was short and changed rapidly after the peak load, indicating that the specimen lost its residual load-bearing capacity within a limited displacement range. Consequently, the post-peak fracture energy was sensitive to local crack propagation, unloading instability, and specimen-to-specimen variability. Although total fracture energy can reflect the overall energy dissipation, it includes the highly scattered post-peak component. Therefore, compared with total fracture energy, pre-peak fracture energy provides a more stable and representative indicator for characterizing the degradation of crack resistance under freeze–thaw cycles and coupled UV aging–freeze–thaw action. Fracture energy was calculated from the vertical load–vertical linear displacement (LLD) curve rather than from the load–crack mouth opening displacement (CMOD) curve.

### Experimental program

To investigate the crack resistance of asphalt concrete under the coupled action of UV aging and freeze-thaw cycles, an SCB test program for coupled UV aging-freeze-thaw damage was designed. With reference to the *Standard Test Methods of Bitumen and Bituminous Mixtures for Highway Engineering* (JTG E20-2011, T0729-2000), the *Technical Specifications for Construction of Highway Asphalt Pavements* (JTG F40-2004), and previous SCB-based freeze–thaw studies^7^, the number of freeze-thaw cycles was set to 0, 5, 10, 15, and 20 to analyze the evolution of crack resistance of UV-aged asphalt concrete under different levels of freeze-thaw action.

**Fig. 2:**
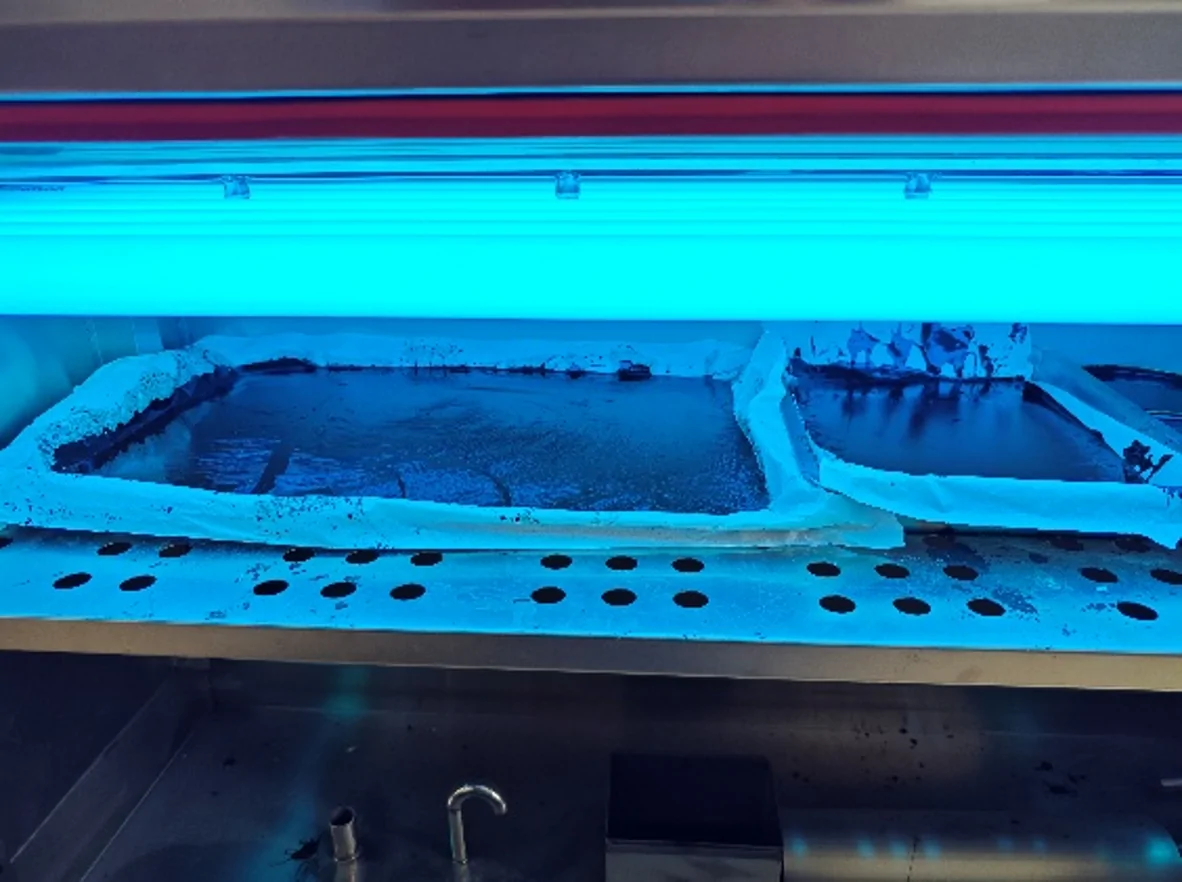
UV aging treatment of asphalt binder.

Considering that UV radiation mainly affects the material surface, direct UV treatment of formed asphalt concrete specimens would make it difficult to ensure consistent aging throughout the specimen interior. Therefore, the asphalt binder was first subjected to UV aging, as shown in Fig. 2, and the aged binder was then used to prepare asphalt mixture specimens so that the influence of UV aging on asphalt concrete performance could be more adequately reflected at the overall material scale. This procedure should be regarded as a homogenized accelerated aging approach. In actual asphalt pavements, UV radiation mainly affects the near-surface asphalt film, and the aging degree decreases with depth. In contrast, the use of pre-aged binder to prepare asphalt mixture results in a more uniform aging state throughout the specimen. This design was adopted to reduce the uncertainty caused by nonuniform surface aging and to evaluate the overall influence of binder aging on the crack resistance of asphalt concrete. Therefore, the obtained results mainly reflect the material-level effect of UV-aged binder on freeze–thaw damage susceptibility, rather than the full depth-dependent aging gradient that occurs in field pavements.

Specifically, the short-term aged asphalt binder was heated to a fluid state and uniformly spread as a thin film with a nominal thickness of approximately 1.8 mm. UV aging was conducted in an accelerated UV-aging chamber equipped with eight UVA-340 fluorescent lamps, which simulate UV radiation with a dominant wavelength of approximately 340 nm. The spectral irradiance was set to 0.89 W/m² at 340 nm, and the chamber temperature was maintained at 60 °C to accelerate photo-oxidative aging while avoiding excessive thermal aging. The UV exposure duration was set as 160 h, and the asphalt film was turned every 40 h to improve aging uniformity.

After UV aging, the aged asphalt binder was used to prepare AC-13 dense-graded asphalt mixture. The asphalt binder was AH-70 base asphalt, and basalt was used as the coarse and fine aggregate. The mixture was designed according to *Technical Specifications for Construction of Highway Asphalt Pavements* (JTG F40-2004), and the optimum asphalt content was determined by the Marshall design method with a target air void content of 4.0%. The raw materials, mixture design, AC-13 gradation, and specimen preparation parameters are summarized in Tables 1 and 2.

**Table 1:** Mixture design and specimen preparation parameters.

Item	Description
Asphalt binder	AH-70 base asphalt
Aggregate type	Basalt aggregate
Mixture type	AC-13 dense-graded asphalt mixture
Design standard	JTG F40-2004
Target air void content	4.0%
Optimum asphalt content	4.9%
Compaction method	Gyratory compaction
Compacted specimen size	150 mm in diameter and 170 mm in height
SCB specimen thickness	50 mm
Notch size	1.5 mm in width and 15 mm in length
Replicates	Three specimens for each freeze–thaw level

**Table 2:** Gradation of AC-13 asphalt mixture.

Sieve size (mm)	16	13.2	9.5	4.75	2.36	1.18	0.6	0.3	0.15	0.075
Passing percentage (%)	100	95	76.5	53	37	26.5	19	13.5	10	6

UV aging was conducted using an accelerated UV-aging chamber equipped with eight UVA-340 fluorescent lamps. The dominant wavelength was approximately 340 nm, the spectral irradiance was 0.89 W/m² at 340 nm, and the chamber temperature was maintained at 60 °C. The short-term aged asphalt binder was heated to a fluid state and spread into a thin film with a nominal thickness of approximately 1.8 mm. The UV exposure duration was set as 160 h, and the asphalt film was turned every 40 h to improve aging uniformity.

The prepared semi-circular specimens were first vacuum-saturated with water at room temperature for 30 min. The freeze–thaw protocol was designed with reference to the *Standard Test Methods of Bitumen and Bituminous Mixtures for Highway Engineering* (JTG E20-2011, T0729-2000). One freeze–thaw cycle consisted of freezing at − 18 °C for 16 h, thawing under full water immersion at 60 °C for 24 h, and subsequent conditioning under full water immersion at 25 °C for 2 h. The specimens were placed in the freeze–thaw apparatus with a spacing of not less than 20 mm between adjacent specimens. The number of freeze–thaw cycles was set as 0, 5, 10, 15, and 20. After the prescribed cycles, the specimens were conditioned at 5 °C for 6 h before NSCB testing.

After conditioning, SCB tests were conducted using a UTM-25 multifunctional material testing system. The SCB test employed a three-point loading configuration with a bottom support span of 120 mm, a test temperature of 5 °C, and a loading rate of 5 mm/min until fracture failure occurred. By comparing the SCB test results of UV-aged asphalt concrete under different numbers of freeze-thaw cycles, the attenuation law of crack resistance of asphalt concrete under coupled UV aging-freeze-thaw action was revealed.

## Results and discussion

**Fig. 3:**
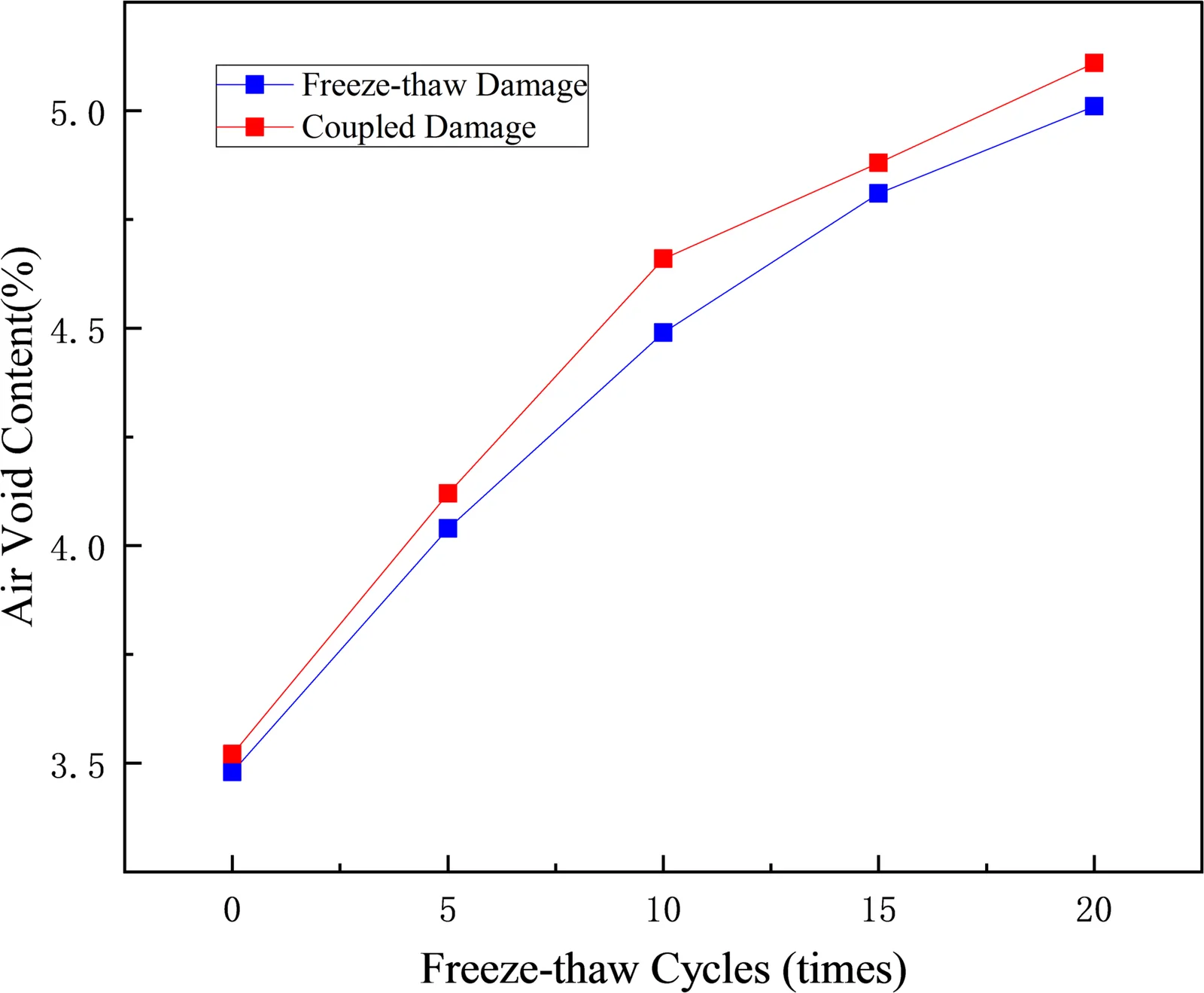
Variation in air void content with freeze-thaw cycles.

**Fig. 4:**
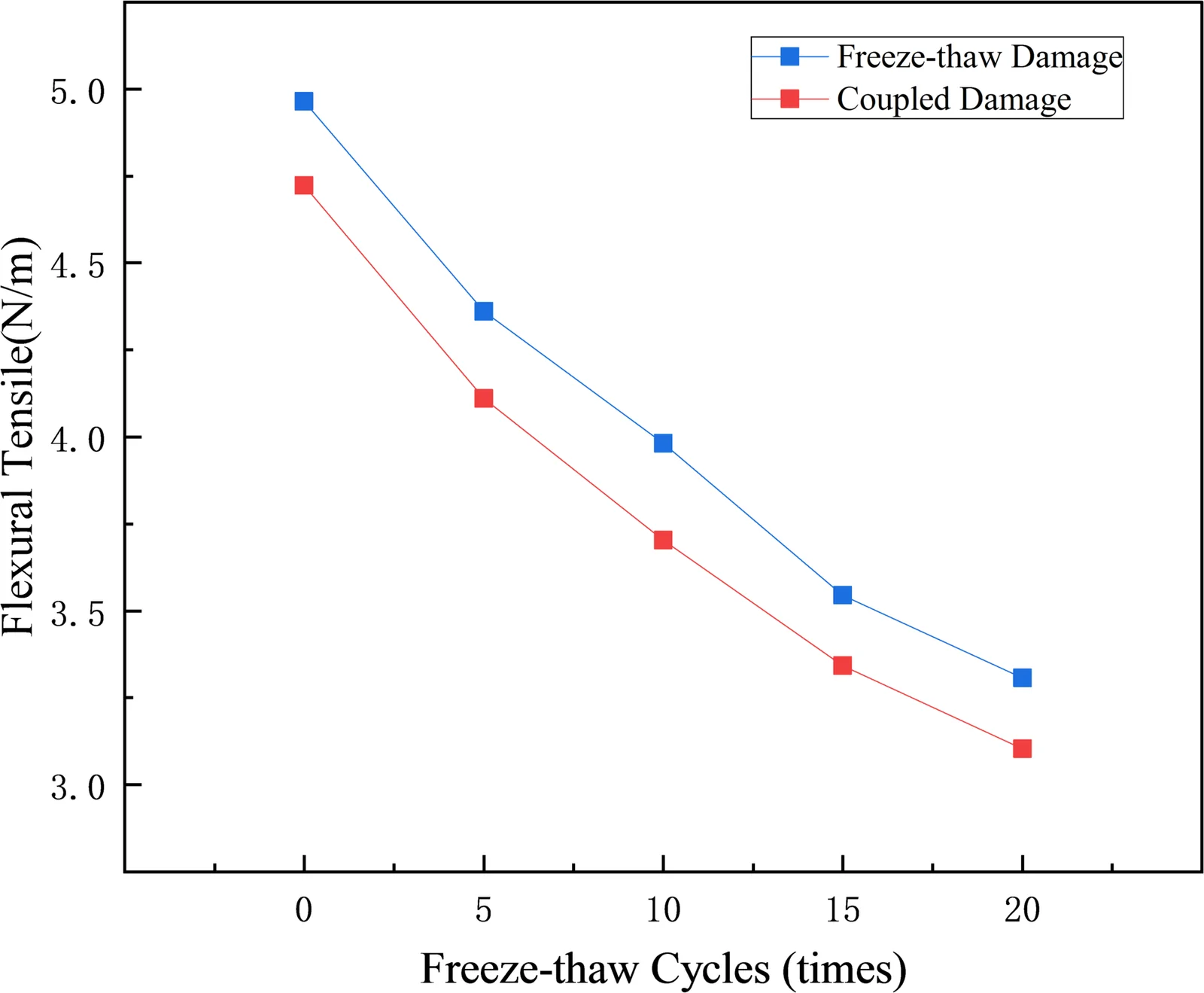
Variation in flexural tensile strength *σ*_*m*_ with freeze-thaw cycles.

**Fig. 5:**
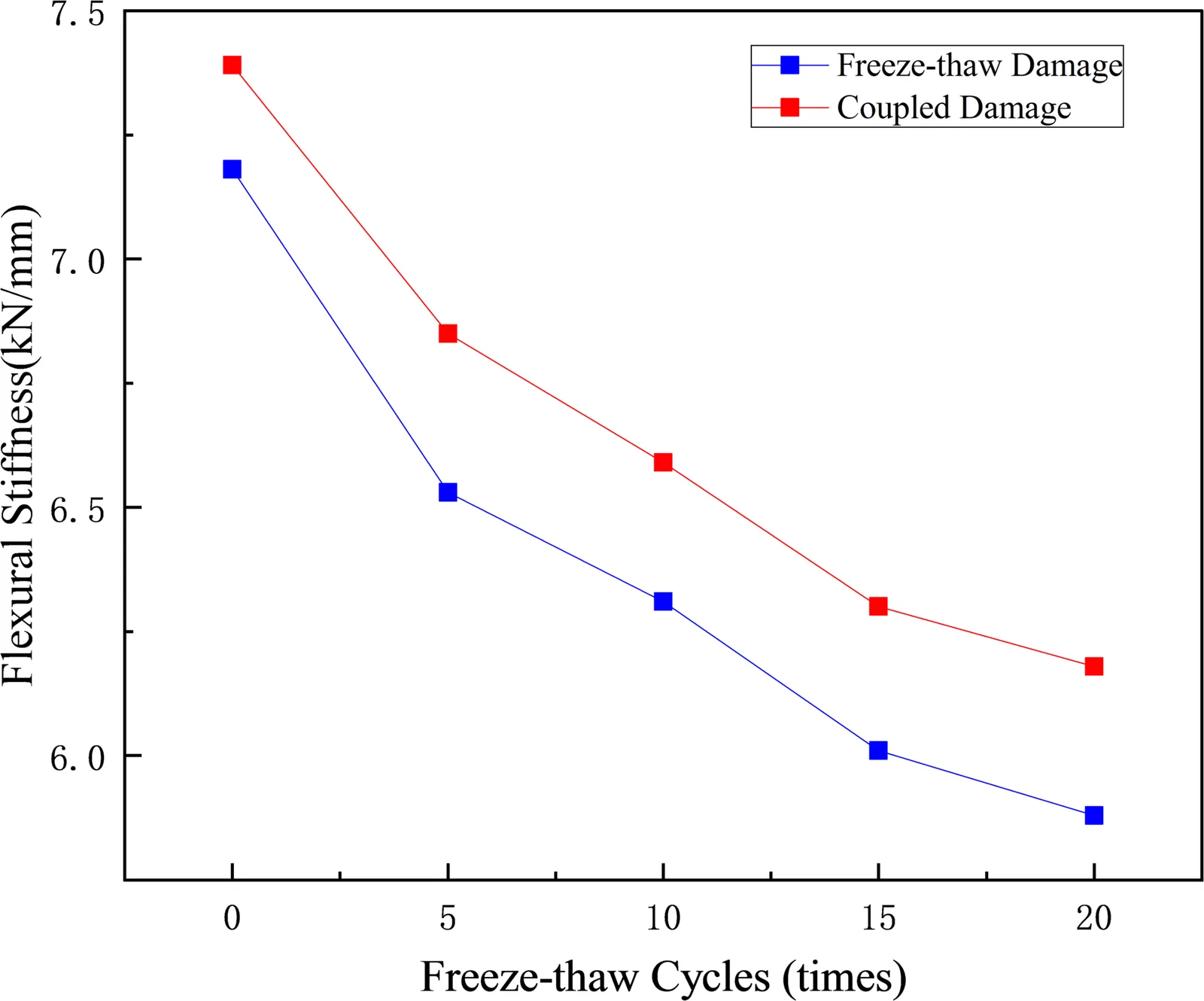
Variation in flexural stiffness *S* with freeze-thaw cycles.

**Fig. 6:**
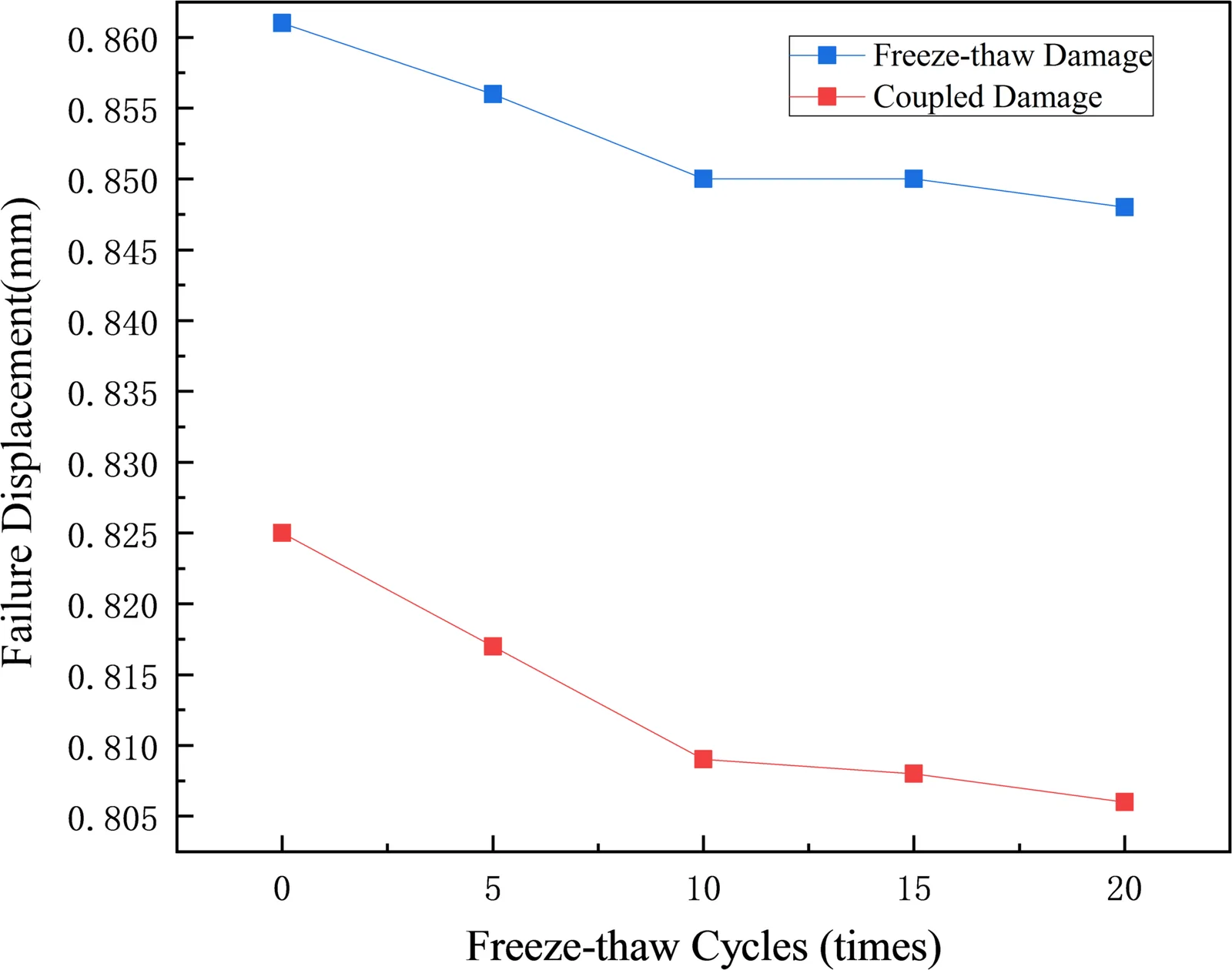
Variation in failure displacement *u*_*0*_ with freeze-thaw cycles.

**Fig. 7:**
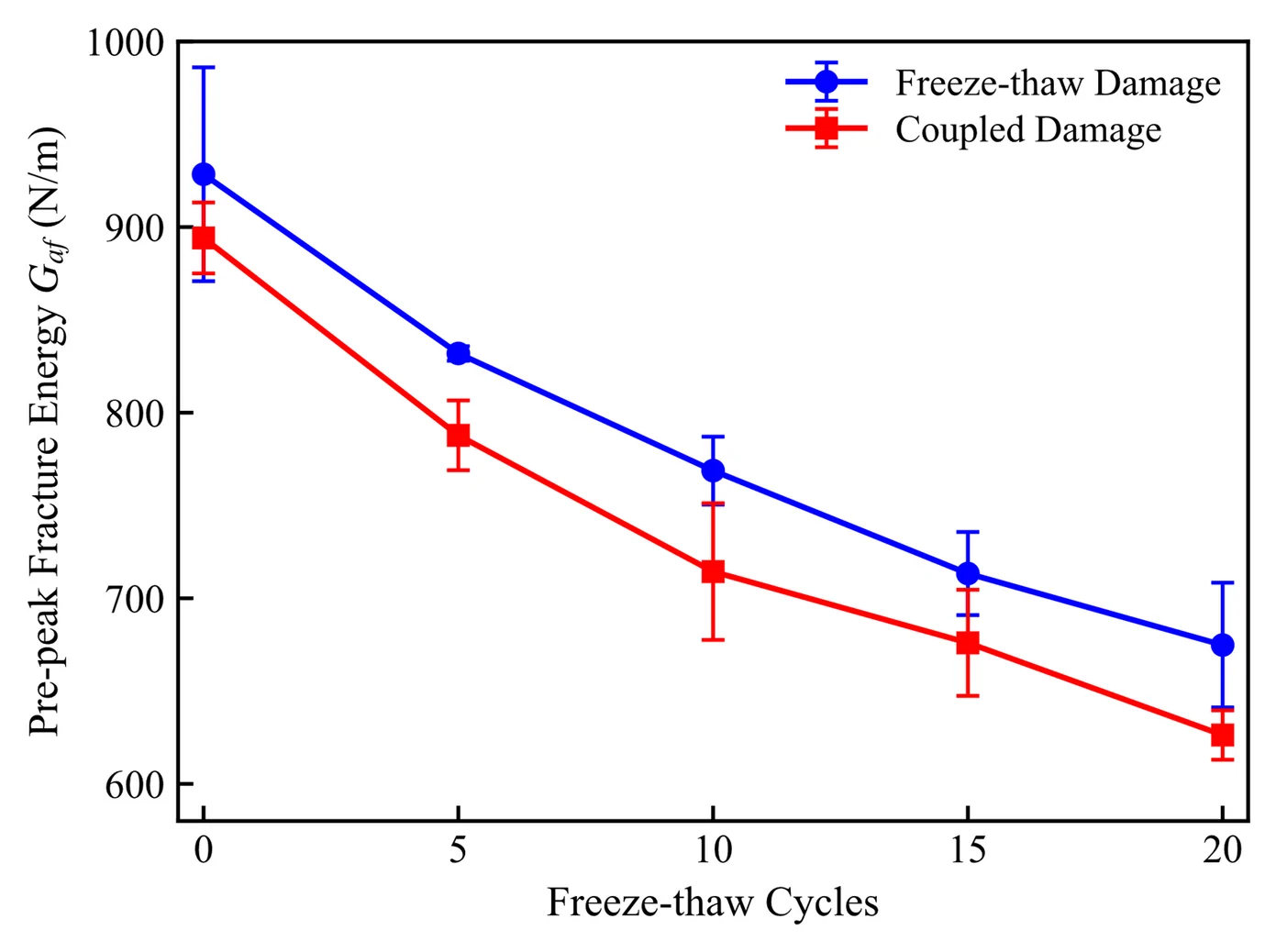
Variation in pre-peak fracture energy *G*_*af*_ with freeze-thaw cycles.

**Fig. 8:**
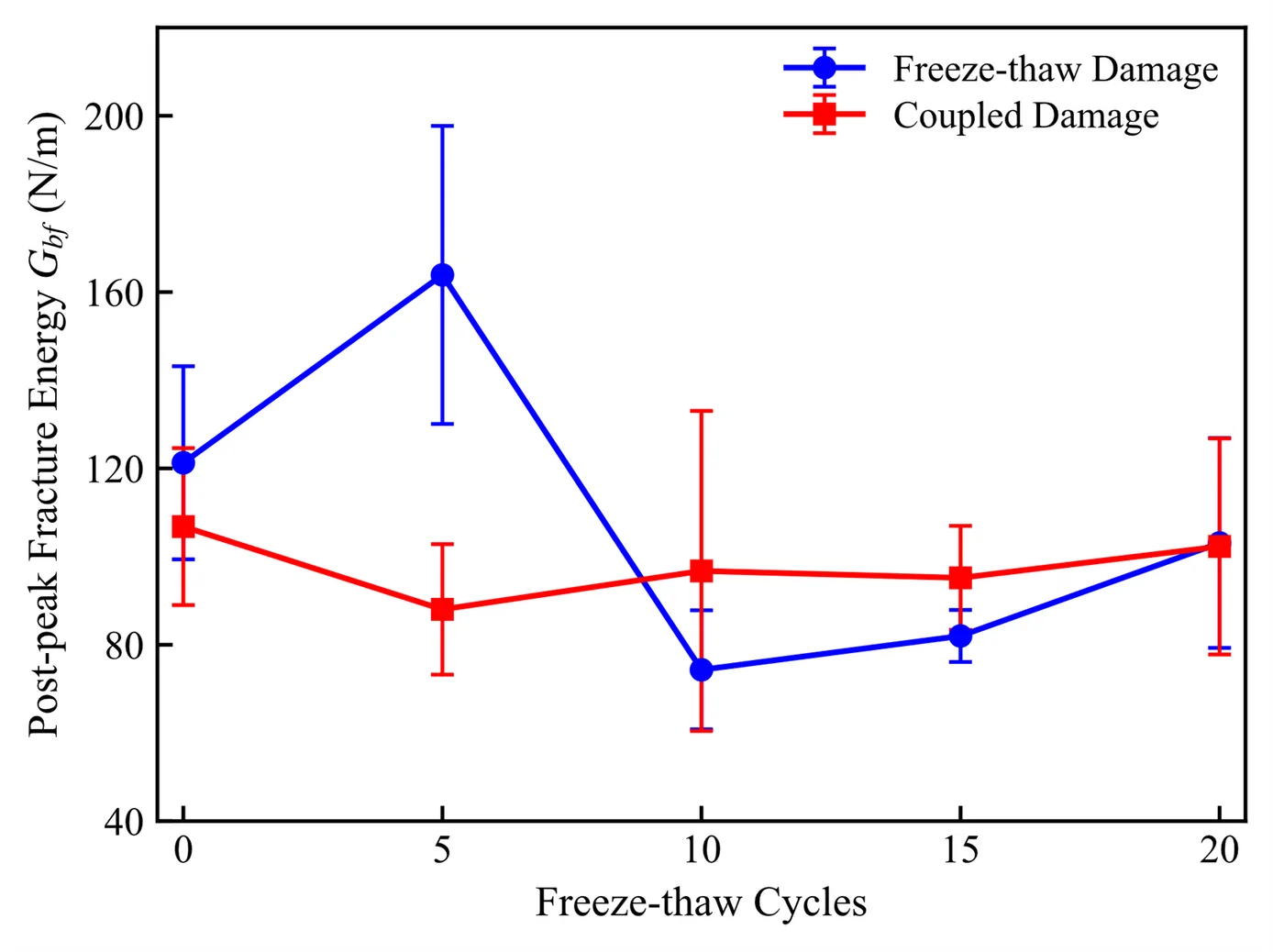
Variation in post-peak fracture energy *G*_*bf*_ with freeze-thaw cycles.

**Fig. 9:**
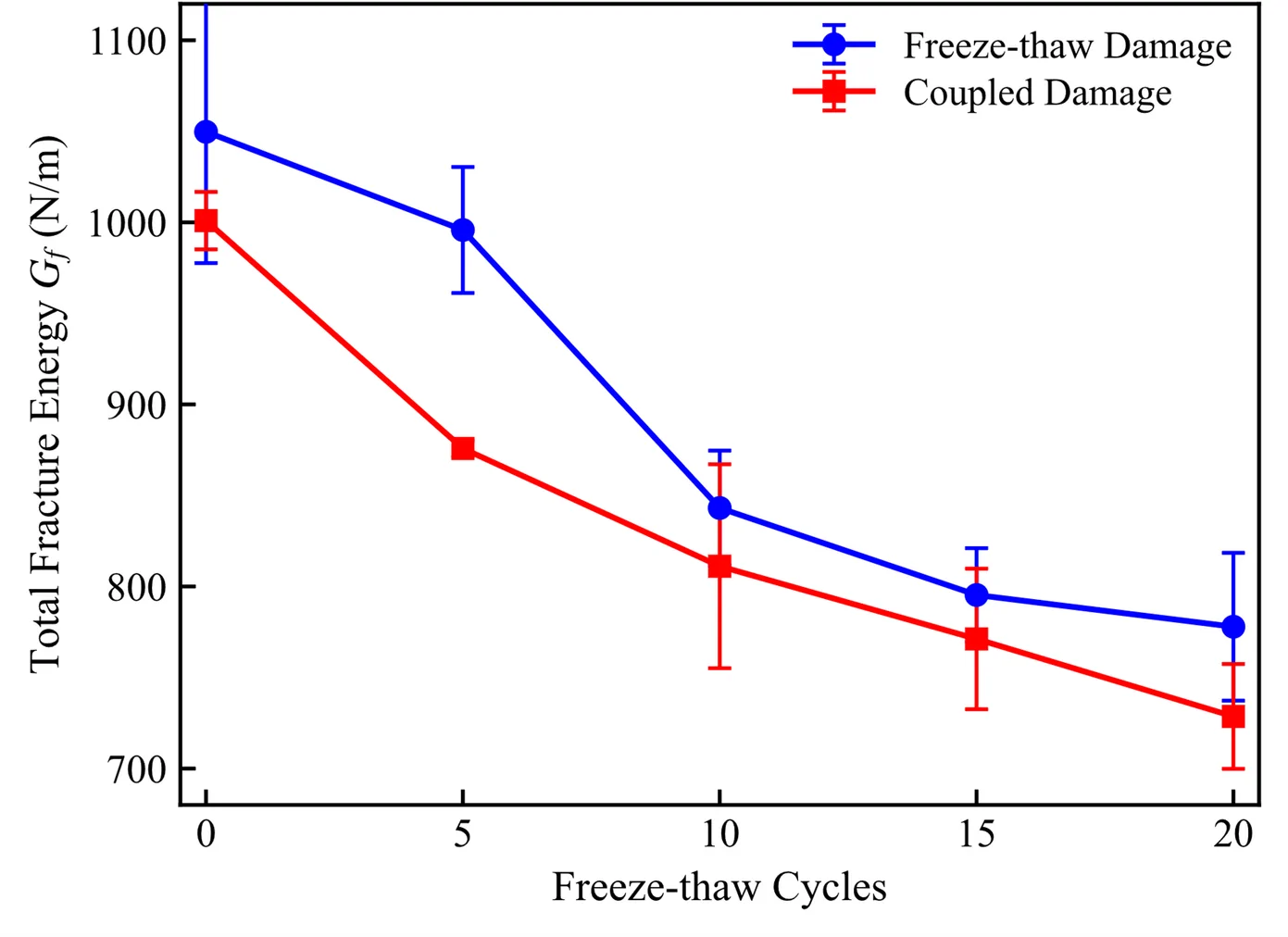
Variation in total fracture energy *G*_*f*_ with freeze-thaw cycles.

For each treatment condition, three replicate specimens were tested. The results in Figs. 3, 4, 5, 6, 7, 8 and 9 are presented as mean values. To evaluate the repeatability of the parallel tests and facilitate comparison of the numerical results, the mean value, standard deviation, and coefficient of variation (CV) of each test indicator are summarized in Table 3. Overall, most indicators show acceptable repeatability, whereas the post-peak fracture energy exhibits relatively larger variability, consistent with its unstable post-peak response under the present NSCB test conditions.

**Table 3:** Mean values, standard deviations, and coefficients of variation of test indicators under different damage conditions.

Condition	F-T cycles	AV (%)	σm (MPa)	S (kN/mm)	u0 (mm)	Gaf (*N*/m)	Gbf (*N*/m)	Gf (*N*/m)
F-T	0	3.48 ± 0.07 (2.01)	4.96 ± 0.10 (2.01)	7.18 ± 1.59 (22.14)	0.86 ± 0.06 (6.97)	928.46 ± 57.60 (6.20)	121.28 ± 21.90 (18.06)	1049.73 ± 72.10 (6.87)
F-T	5	4.04 ± 0.07 (1.73)	4.36 ± 0.13 (2.98)	6.53 ± 0.38 (5.82)	0.86 ± 0.02 (2.34)	831.91 ± 3.90 (0.47)	163.89 ± 33.80 (20.62)	995.80 ± 34.60 (3.47)
F-T	10	4.49 ± 0.14 (3.12)	3.98 ± 0.06 (1.51)	6.31 ± 0.97 (15.37)	0.85 ± 0.04 (4.71)	768.78 ± 18.30 (2.38)	74.33 ± 13.50 (18.16)	843.11 ± 31.50 (3.74)
F-T	15	4.81 ± 0.11 (2.29)	3.54 ± 0.11 (3.10)	6.01 ± 0.37 (6.16)	0.85 ± 0.03 (3.53)	713.33 ± 22.40 (3.14)	82.00 ± 5.90 (7.20)	795.33 ± 25.70 (3.23)
F-T	20	5.01 ± 0.22 (4.39)	3.31 ± 0.07 (2.12)	5.88 ± 0.44 (7.48)	0.85 ± 0.02 (2.36)	674.80 ± 33.60 (4.98)	103.09 ± 23.80 (23.09)	777.89 ± 40.60 (5.22)
Coupled	0	3.52 ± 0.09 (2.56)	4.72 ± 0.09 (1.91)	7.39 ± 0.13 (1.76)	0.82 ± 0.01 (1.21)	894.14 ± 19.10 (2.14)	106.81 ± 17.80 (16.67)	1000.95 ± 15.80 (1.58)
Coupled	5	4.12 ± 0.08 (1.94)	4.11 ± 0.06 (1.46)	6.85 ± 0.40 (5.84)	0.82 ± 0.02 (2.45)	787.78 ± 18.80 (2.39)	88.03 ± 14.80 (16.81)	875.81 ± 4.50 (0.51)
Coupled	10	4.66 ± 0.10 (2.15)	3.70 ± 0.08 (2.16)	6.59 ± 0.73 (11.08)	0.81 ± 0.04 (4.94)	714.37 ± 36.80 (5.15)	96.75 ± 36.30 (37.52)	811.12 ± 56.00 (6.90)
Coupled	15	4.88 ± 0.10 (2.05)	3.34 ± 0.03 (0.90)	6.30 ± 0.29 (4.60)	0.81 ± 0.01 (1.24)	676.00 ± 28.60 (4.23)	95.18 ± 11.80 (12.40)	771.18 ± 38.60 (5.01)
Coupled	20	5.11 ± 0.12 (2.35)	3.10 ± 0.07 (2.26)	6.18 ± 0.17 (2.75)	0.81 ± 0.004 (0.50)	626.31 ± 13.30 (2.12)	102.30 ± 24.50 (23.95)	728.61 ± 28.80 (3.95)

Note: AV = air void content; *σ*_*m*_ = flexural tensile strength; *S* = flexural stiffness; *u*_*0*_ = failure displacement; *G*_*af*_ = pre-peak fracture energy; *G*_*bf*_ = post-peak fracture energy; *G*_*f*_ = total fracture energy. Values in parentheses are CV (%).

### Effect of freeze-thaw cycles on air void content

Air void content is an important indicator of the compactness of the internal structure of asphalt concrete. During freeze-thaw cycling, the frost-heaving effect induced by repeated freezing and thawing of moisture in internal pores promotes further expansion and coalescence of existing voids and microcracks, thereby causing internal structural damage and adversely affecting crack resistance. To analyze the influence of freeze-thaw cycling and coupled UV aging-freeze-thaw action on the internal structure of asphalt concrete, the air void content of semi-circular specimens under different treatment conditions was determined by the saturated-surface-dry method. The results are shown in Fig. 3.

Figure 3 shows that the air void content of asphalt concrete generally increases with the number of freeze-thaw cycles, indicating that freeze-thaw action continuously weakens the compactness of the internal structure. Compared with the specimens subjected only to freeze-thaw cycles, the specimens exposed to coupled UV aging-freeze-thaw damage show slightly higher overall air void content, although the increment is relatively limited. After 5, 10, 15, and 20 freeze-thaw cycles, the air void content of the coupled-damage specimens increased by 1.2%, 2.0%, 3.8%, and 2.0%, respectively, relative to the specimens subjected to freeze-thaw cycles alone. This indicates that freeze-thaw cycling remains the dominant factor governing the evolution of the void structure and internal damage of asphalt concrete, while UV aging further aggravates this damage process to a certain extent.

### SCB test results

#### Flexural tensile strength

Flexural tensile strength reflects the ultimate load-bearing capacity of asphalt concrete under loading, and its variation can be used to characterize the degradation of crack resistance. The flexural tensile strength of semi-circular asphalt concrete specimens subjected to different numbers of freeze-thaw cycles and its variation law are shown in Fig. 4.

Figure 4 shows that the flexural tensile strength of the semi-circular asphalt concrete specimens continuously decreases as the number of freeze-thaw cycles increases, indicating that the specimens fail at lower load levels and their load-bearing capacity is clearly weakened. This demonstrates that freeze-thaw cycling continuously intensifies internal damage in asphalt concrete and reduces the material’s ability to resist crack initiation and propagation. Compared with the specimens subjected only to freeze-thaw cycles, the flexural tensile strength of the specimens exposed to coupled UV aging-freeze-thaw damage is further reduced under the same freeze-thaw condition, with reductions of 4.87%, 5.73%, 6.98%, 5.67%, and 6.14%, respectively. This indicates that UV aging aggravates freeze-thaw damage in asphalt concrete. This deterioration can be interpreted from the chemical and interfacial effects of UV aging on the asphalt binder. Binder-level characterization showed that UV aging promoted the transformation of light fractions into heavier fractions, with aromatic components gradually converting into asphaltenes. Meanwhile, the carbonyl and sulfoxide indices increased with UV-aging duration, indicating enhanced photo-oxidation and molecular polarity. These chemical changes increased binder stiffness and brittleness while reducing its cohesive capacity. Pull-off adhesion results further indicated that UV aging weakened the asphalt–aggregate adhesive strength. Under subsequent freeze–thaw cycles, the aged and embrittled asphalt film was less able to accommodate frost-heave deformation and moisture-induced interfacial disturbance, making the asphalt–aggregate interface more susceptible to debonding, void expansion, and microcrack propagation. Consequently, the coupled-damage specimens exhibited lower flexural tensile strength and fracture energy than the specimens subjected to freeze–thaw cycles alone.

In addition, the fitting results in Fig. 4 show that, for both freeze-thaw damage alone and coupled UV aging-freeze-thaw damage, the flexural tensile strength of asphalt concrete exhibits an obvious approximately linear decreasing trend with increasing freeze-thaw cycles, and the linear regression provides a good fit. This indicates that flexural tensile strength responds regularly to freeze-thaw damage.

#### Flexural stiffness

Flexural stiffness is used to characterize the ability of semi-circular asphalt concrete specimens to resist deformation and cracking at the initial loading stage. Its variation is shown in Fig. 5.

Figure 5 shows that the flexural stiffness of asphalt concrete generally decreases with increasing freeze-thaw cycles, indicating that freeze-thaw cycling continuously weakens the deformation resistance of the material at the early stage of loading. This trend is basically consistent with that of flexural tensile strength, further demonstrating the significant deterioration of mechanical properties caused by freeze-thaw damage. Unlike flexural tensile strength, however, under the same freeze-thaw condition the specimens subjected to coupled UV aging-freeze-thaw damage exhibit overall higher flexural stiffness than the specimens exposed only to freeze-thaw cycles, and the difference between the two gradually increases with the number of freeze-thaw cycles. This indicates that although UV aging weakens the overall crack resistance of asphalt concrete, it can increase the initial stiffness of the material to a certain extent. The reason is that UV aging hardens the asphalt binder and increases the material modulus, thereby enhancing the resistance of the specimen to deformation at the initial loading stage. As freeze-thaw cycling progresses, the air void content of asphalt concrete continues to increase, while interfacial damage and aggregate spalling gradually intensify, causing the internal structure to become looser. Under these conditions, the stiffening effect of the aged binder on flexural stiffness becomes more apparent, so that the rate of flexural stiffness reduction in the coupled-damage specimens is lower than that in the unaged specimens. Therefore, in terms of flexural stiffness, UV aging mainly causes asphalt concrete to shift from a more ductile to a more brittle state, namely a higher initial deformation resistance but poorer deformation compatibility and reduced subsequent crack resistance.

#### Failure displacement

Failure displacement is an important indicator of the deformation capacity of a material before fracture, and to a certain extent reflects the ability of asphalt concrete to resist crack propagation. The variation in failure displacement of the specimens subjected to different numbers of freeze-thaw cycles is shown in Fig. 6.

Figure 6 shows that, under freeze-thaw cycles alone, the failure displacement of asphalt concrete specimens varies only slightly and does not exhibit a clear decreasing trend overall. For the specimens without UV aging, the failure displacement after 20 freeze-thaw cycles decreases by only 1.47% compared with that at 0 cycles, indicating that freeze-thaw cycling has a relatively limited influence on this index. By contrast, the failure displacement of the specimens subjected to coupled UV aging-freeze-thaw damage is lower than that of the unaged specimens at all freeze-thaw levels and shows a slight downward trend with increasing freeze-thaw cycles. Specifically, the failure displacement decreases from 0.825 mm at 0 freeze-thaw cycles to 0.806 mm after 20 freeze-thaw cycles, corresponding to a reduction of 2.23%. This indicates that UV aging weakens the deformation compatibility of asphalt concrete, causing fracture failure to occur at smaller displacements and thereby aggravating the deterioration of crack resistance.

Overall, failure displacement is not sufficiently sensitive to freeze-thaw damage alone, but it can still, to some extent, reflect the increase in material embrittlement when characterizing the coupled damage effect involving UV aging.

#### Fracture energy

According to Eq. (3), the pre-peak fracture energy *G*_*af*_, post-peak fracture energy *G*_*bf*_, and total fracture energy *G*_*f*_ of the semi-circular specimens were calculated^26^. The variation of these fracture-energy indices with the number of freeze-thaw cycles is shown in Figs. 7, 8 and 9, respectively. Overall, the fracture-energy indices exhibit greater scatter than flexural tensile strength, flexural stiffness, and failure displacement.

Overall, compared with flexural tensile strength, flexural stiffness, and failure displacement, the fracture-energy indices show relatively larger data dispersion. Among them, the pre-peak fracture energy *G*_*af*_ exhibits a clear decreasing trend with increasing freeze-thaw cycles and can better reflect the deterioration of the crack resistance of asphalt concrete during crack initiation and stable propagation. For the specimens without UV aging, the pre-peak fracture energy decreases by 10.40%, 17.20%, 23.17%, and 27.32% after 5, 10, 15, and 20 freeze-thaw cycles, respectively. For the specimens subjected to coupled UV aging-freeze-thaw damage, the corresponding reductions are 11.90%, 20.11%, 24.40%, and 29.95%. Under the same freeze-thaw condition, the pre-peak fracture energy of the coupled-damage specimens is further reduced by 3.70%, 5.30%, 7.08%, 5.23%, and 7.18% relative to that of the specimens subjected only to freeze-thaw cycles, indicating that UV aging aggravates the deterioration of crack resistance caused by freeze-thaw cycling. The fitting results in Fig. 7 also show a good linear correlation between pre-peak fracture energy and the number of freeze-thaw cycles, indicating that this index is sensitive to both freeze-thaw damage and coupled damage.

The variation in post-peak fracture energy *G*_*bf*_ is shown in Fig. 8. It can be seen that *G*_*bf*_ is much smaller than *G*_*af*_, indicating that asphalt concrete has limited residual load-bearing capacity after crack formation and that once a crack is generated, the specimen rapidly becomes unstable and fails within a short period. Meanwhile, *G*_*bf*_ does not exhibit a clear monotonic trend with the number of freeze-thaw cycles and shows large scatter among specimens. This is because, under the test conditions of this study, the descending branch of the load-displacement curve is short and changes rapidly, and the unloading responses differ markedly among specimens, resulting in insufficient stability of the post-peak fracture energy. Therefore, post-peak fracture energy is not suitable as a primary indicator for evaluating the crack resistance of asphalt concrete.

The total fracture energy *G*_*f*_ is the sum of pre-peak and post-peak fracture energy, and its variation is shown in Fig. 9. Because pre-peak fracture energy dominates the total fracture energy, the variation trend of *G*_*f*_ with freeze-thaw cycles is basically consistent with that of *G*_*af*_, namely an overall decreasing trend, and the total fracture energy of the specimens subjected to coupled UV aging-freeze-thaw damage is smaller than that of the specimens without UV aging under the same freeze-thaw condition. However, because *G*_*f*_ also includes the highly scattered post-peak fracture energy component, its correlation and stability with respect to freeze-thaw cycles are weaker than those of pre-peak fracture energy. Comprehensive comparison shows that pre-peak fracture energy can more accurately and stably characterize the attenuation law of crack resistance of asphalt concrete under freeze-thaw cycles and coupled UV aging-freeze-thaw action.

### Damage index analysis of performance indicators

Because air void content and the crack-resistance indices differ substantially in dimension and numerical range, and their sensitivities to freeze–thaw cycles are inconsistent, direct comparison of raw data cannot intuitively reflect the damage extent represented by different indicators. To achieve a unified evaluation, the damage index (DI) was introduced and defined as the ratio of the absolute difference between the value *N*_*i*_ of a given indicator after the *i*th freeze–thaw cycle and its initial value *N*_*0*_ to the initial value, as expressed in Eq. (4). It should be noted that different indicators have different physical deterioration directions. For air void content, an increase indicates a reduction in internal compactness and more severe structural damage. In contrast, for flexural tensile strength, flexural stiffness, failure displacement, pre-peak fracture energy, and total fracture energy, a decrease indicates degradation of load-bearing capacity, deformation capacity, or energy-dissipation capacity. Therefore, the damage index in this study represents the normalized magnitude of deterioration for each parameter, rather than the algebraic increase or decrease of the raw value. After normalization, this indicator can be used to compare the responses of different performance parameters to freeze–thaw action.

4 $$\:\begin{array}{c}DI=\frac{\left|{N}_{i}-{N}_{0}\right|}{{N}_{0}}\times\:100\% \end{array}$$

**Fig. 10:**
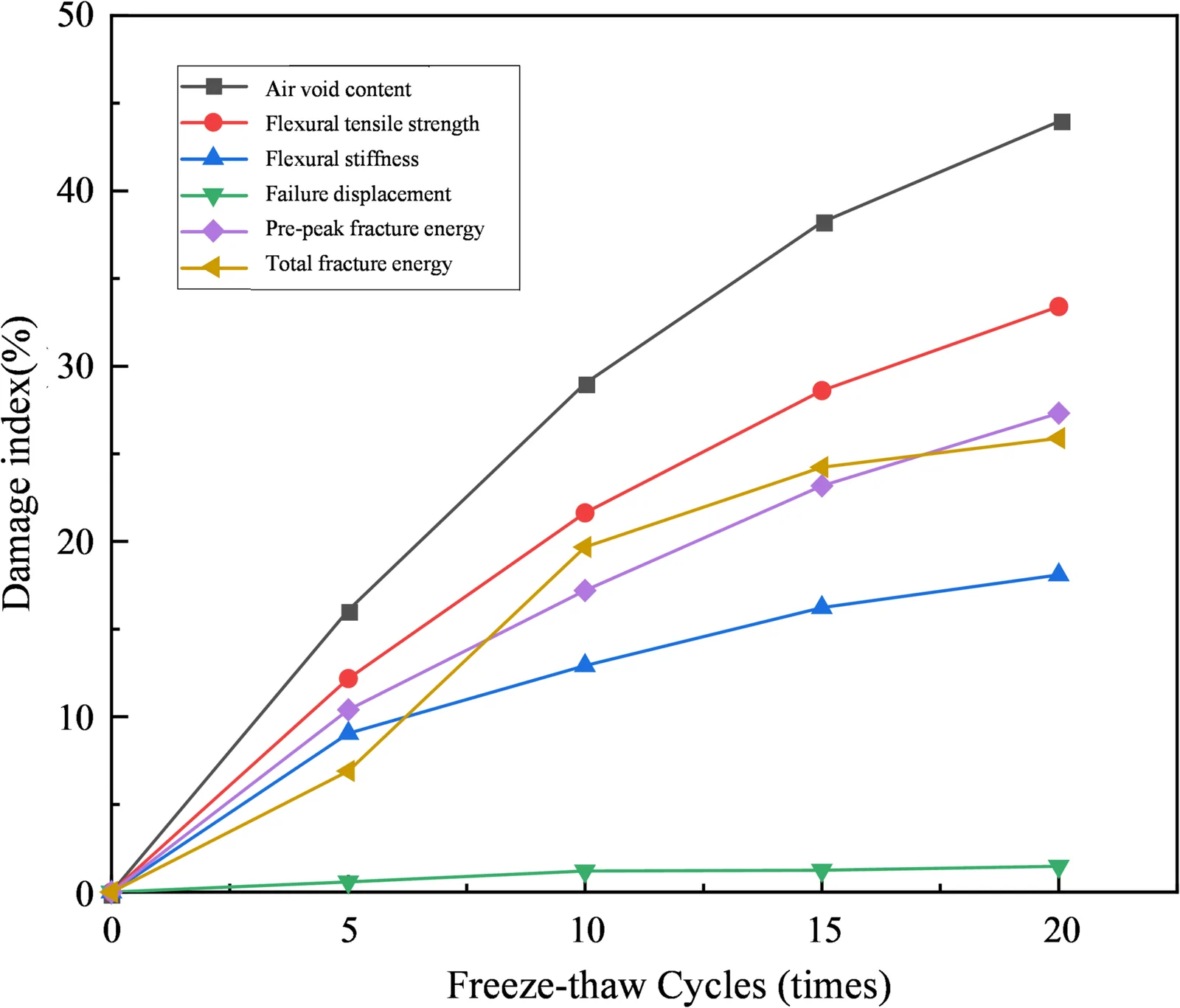
Effect of freeze-thaw cycles on the damage indices of different indicators.

**Fig. 11:**
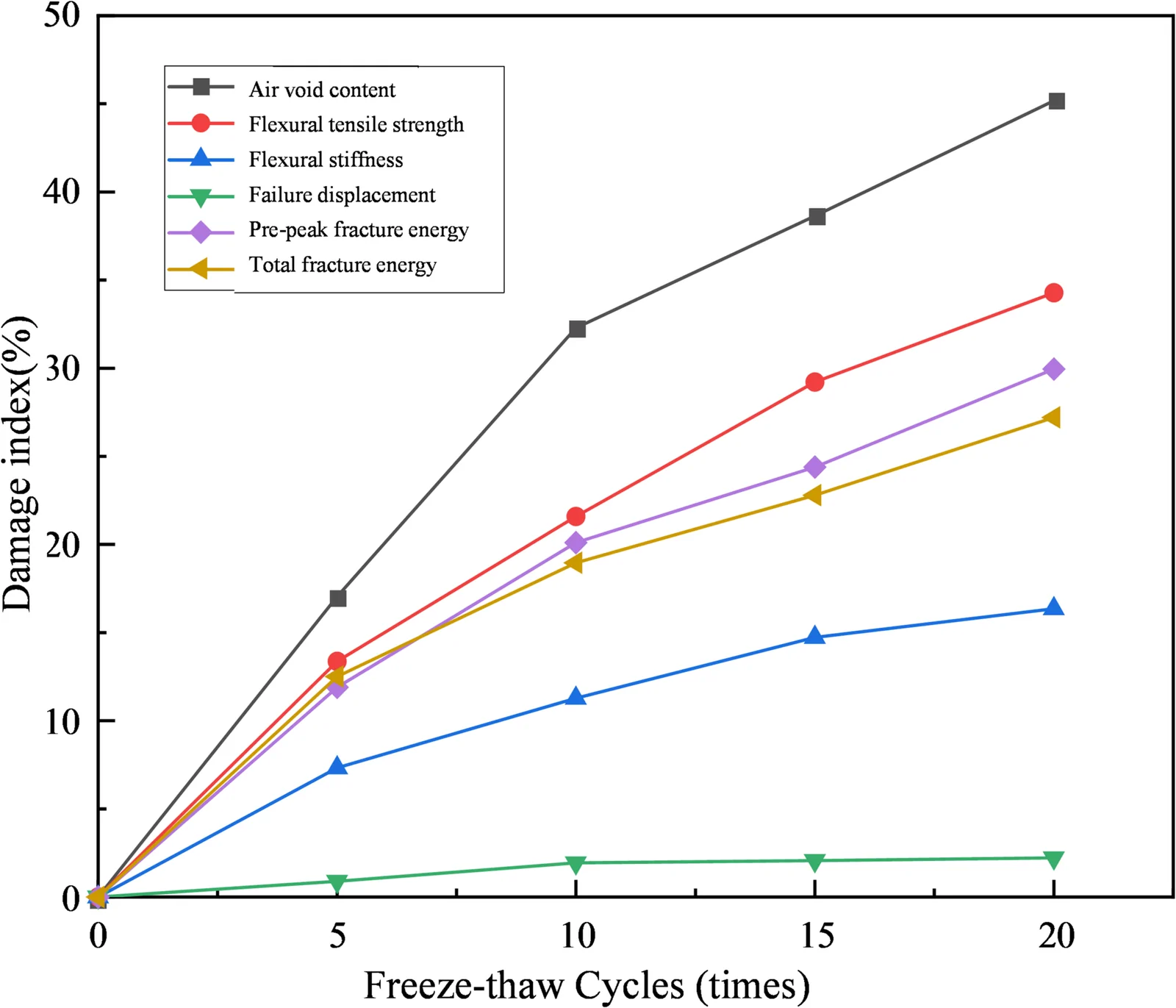
Effect of coupled action on the damage indices of different indicators.

According to Eq. (4), the damage indices of air void content, flexural tensile strength, flexural stiffness, failure displacement, pre-peak fracture energy, and total fracture energy were calculated, respectively. Considering the large scatter and unclear variation law of post-peak fracture energy, it was not included in the damage-index analysis. The relevant results are shown in Figs. 10 and 11.

The results show that the sensitivities of the different indicators to freeze-thaw cycles differ markedly. Among them, the damage index of flexural tensile strength shows the largest increase, reaching 46.07% and 50.06% after 20 freeze-thaw cycles for the freeze-thaw-alone group and the coupled-damage group, respectively. The damage indices of pre-peak fracture energy, total fracture energy, and air void content are relatively close, whereas the damage index of failure displacement remains small throughout and is still below 5% after 20 freeze-thaw cycles, indicating its relatively limited response to freeze-thaw damage. Comparison of the two damage conditions shows that, except for flexural stiffness, the damage indices of all indicators under coupled damage are generally higher than those under freeze-thaw action alone, indicating that UV aging accelerates the performance degradation of asphalt concrete under freeze-thaw cycles. Although UV aging increases binder stiffness and leads to some enhancement in flexural stiffness, the continuous reductions in flexural tensile strength and fracture energy indicate that this stiffening effect is insufficient to offset the adverse influence of material embrittlement and microcrack propagation.

In addition, the variation of the damage indices of all indicators with freeze-thaw cycles generally exhibits a nonlinear feature characterized by rapid growth in the early stage and gradual flattening in the later stage. Therefore, a Logistic function was used to fit the evolution law of the damage indices^27^. The functional expression is given in Eq. (5).

5 $$\:\begin{array}{c}L\left(x\right)=a\left(1-\frac{1}{1+{\left(\frac{x}{b}\right)}^{2}}\right) \end{array}$$

**Fig. 12:**
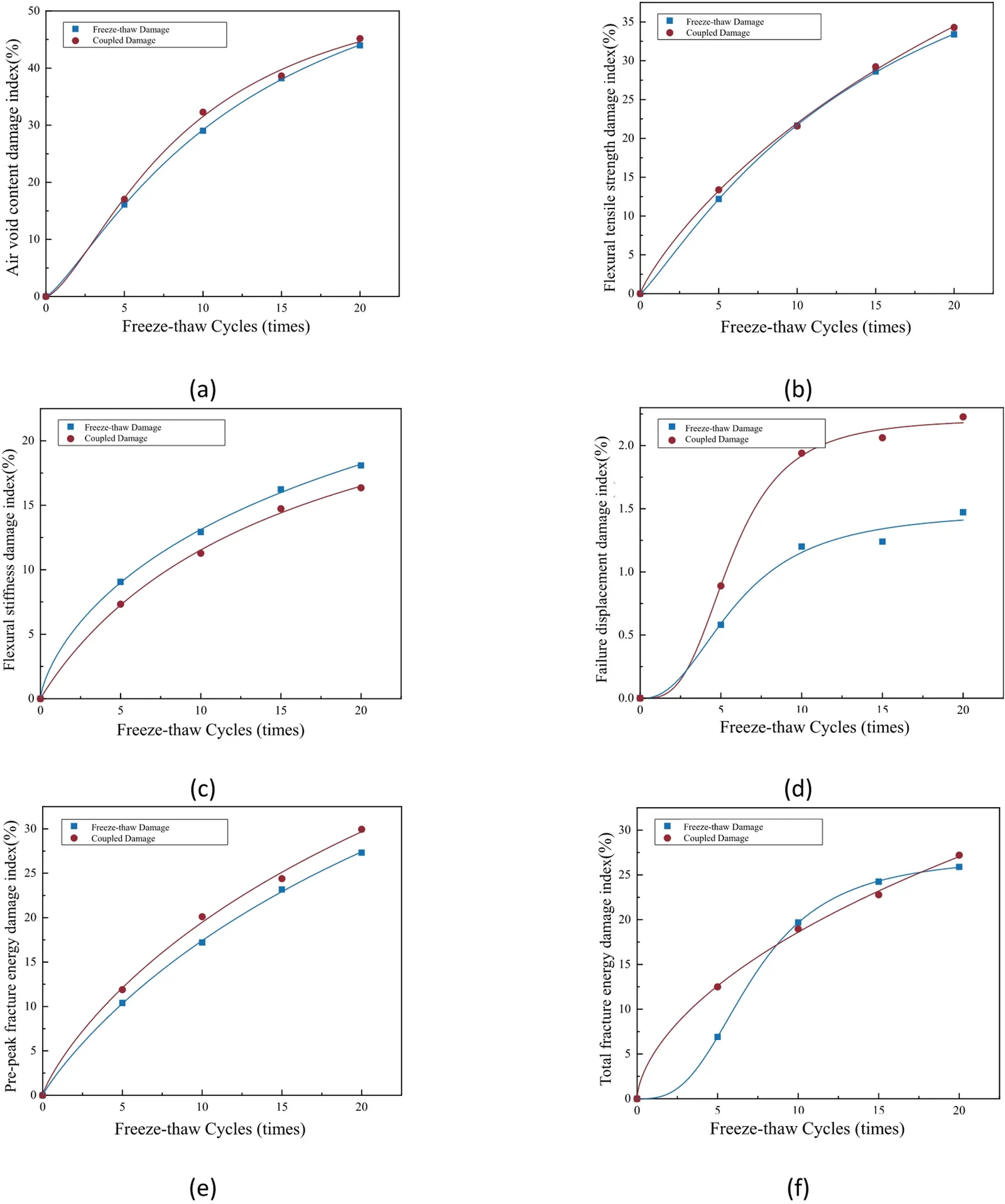
Evolution of damage indices of different parameters with freeze-thaw cycles. (a) Air void content damage index, (b) flexural tensile strength damage index, (c) flexural stiffness damage index, (d) failure displacement damage index, (e) pre-peak fracture energy damage index, (f) total fracture energy damage index.

The fitted curves of the damage indices are shown in Fig. 12.

The corresponding Logistic fitting parameters and exact R^2^ values are summarized in Table 4. In Eq. (5), parameter *a* represents the upper asymptotic value of the damage index, reflecting the limiting damage level under continued freeze–thaw action; parameter *b* is related to the horizontal position of the curve and affects the onset of rapid damage accumulation; parameter *c* controls the growth rate of the damage index.

**Table 4:** Logistic fitting parameters and R^2^ values for damage indices.

Damage condition	Indicator	a	b	c	*R* ^2^
Freeze–thaw damage	Air void content	58.08	8.91	1.49	0.993
Freeze–thaw damage	Flexural tensile strength	59.03	15.90	1.17	0.999
Freeze–thaw damage	Flexural stiffness	46.18	36.66	0.71	0.998
Freeze–thaw damage	Failure displacement	1.49	5.95	2.39	0.955
Freeze–thaw damage	Pre-peak fracture energy	78.78	39.85	0.91	0.999
Freeze–thaw damage	Total fracture energy	27.13	7.20	2.94	0.999
Coupled damage	Air void content	68.49	12.62	1.28	0.999
Coupled damage	Flexural tensile strength	112.78	51.89	0.86	0.998
Coupled damage	Flexural stiffness	29.87	16.13	0.97	0.996
Coupled damage	Failure displacement	2.22	5.64	3.26	0.992
Coupled damage	Pre-peak fracture energy	91.63	48.67	0.83	0.992
Coupled damage	Total fracture energy	208.95	465.51	0.61	0.997

As shown in Table 3, the R² values of all fitted curves are higher than 0.95, indicating that the Logistic function can effectively describe the nonlinear cumulative evolution of the damage indices with increasing freeze–thaw cycles. The differences in fitted parameters further indicate that the damage accumulation rate and limiting damage level vary among indicators and damage conditions.

### Correlation analysis of damage indicators

**Fig. 13:**
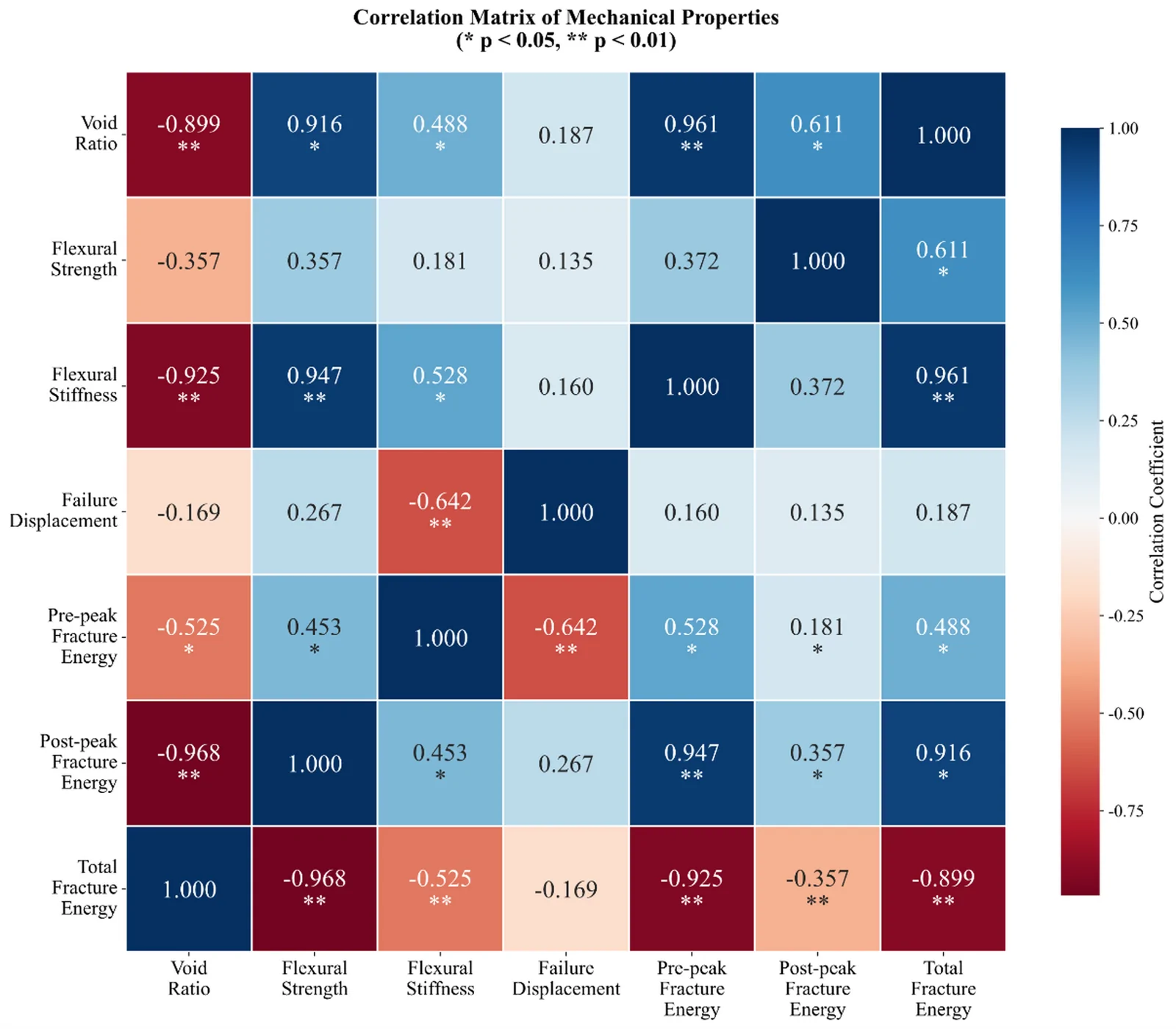
Pearson correlation heatmap (*n = 30*; * indicates *p* < 0.05, and ** indicates *p* < 0.01, two-tailed test).

To further analyze the relationships between air void content and the crack-resistance indices, Pearson correlation analysis was performed on seven parameters, namely air void content, flexural tensile strength, flexural stiffness, failure displacement, pre-peak fracture energy, post-peak fracture energy, and total fracture energy. The analysis was conducted using the full dataset obtained from the two damage conditions, five freeze–thaw levels, and three replicate specimens for each condition, giving a total sample size of *n* = 30. Before Pearson correlation analysis, the normality of the selected indicators was checked. The significance of the correlation coefficients was evaluated using a two-tailed test, with * and ** denoting significance at the 0.05 and 0.01 levels, respectively. A Pearson correlation heatmap with significance marks was drawn using SPSS software, as shown in Fig. 13.

The results show that air void content is negatively correlated with all crack-resistance indices except failure displacement. Considering the relatively limited sample size, the Pearson correlation results should be interpreted as statistical evidence for the consistency and sensitivity of the selected indicators rather than as proof of a deterministic causal relationship. Therefore, the conclusions regarding key indicators were drawn by jointly considering the variation trends, damage-index results, and correlation significance. The strength of correlation follows the order: flexural tensile strength > pre-peak fracture energy > total fracture energy > flexural stiffness, indicating that an increase in air void content leads to a decrease in the crack resistance of asphalt concrete, among which flexural tensile strength is the most sensitive to internal damage. Among the crack-resistance indices, flexural tensile strength shows a significant positive correlation with pre-peak fracture energy, with a correlation coefficient of 0.947, indicating good consistency between the two in characterizing the crack resistance of the material. By contrast, the correlations of flexural stiffness and failure displacement with the other indices are generally insignificant. The correlation of post-peak fracture energy with the other indices is also weak, indicating insufficient stability and suggesting that it is not suitable as a primary evaluation indicator.

## Conclusions

Based on SCB tests, this study investigated the evolution of the void structure and crack resistance of asphalt concrete under coupled UV aging and freeze-thaw cycles. The main conclusions are as follows:

1. As the number of freeze-thaw cycles increased, the air void content of asphalt concrete gradually increased, indicating that freeze-thaw cycling progressively weakened the compactness of the internal structure. Compared with the specimens subjected to freeze-thaw action alone, the specimens exposed to coupled UV aging and freeze-thaw damage showed slightly higher overall air void content, suggesting that UV aging may further aggravate internal structural damage under freeze-thaw action.
2. Freeze-thaw cycling led to a clear deterioration in the crack resistance of asphalt concrete. In particular, flexural tensile strength and pre-peak fracture energy decreased with the number of freeze-thaw cycles, and the corresponding values of the coupled-damage specimens were consistently lower than those of the specimens subjected only to freeze-thaw cycles under the tested conditions, indicating that UV aging further intensified the deterioration of load-bearing capacity and crack resistance. By contrast, although flexural stiffness also showed an overall decreasing trend, it remained slightly higher in the coupled-damage specimens than in the specimens subjected only to freeze-thaw cycles. Failure displacement changed only slightly, and post-peak fracture energy showed relatively large scatter, suggesting that neither index is suitable as a primary evaluation indicator in this study.
3. Damage index analysis showed that flexural tensile strength was the most sensitive index to freeze-thaw and coupled damage, followed by pre-peak fracture energy, total fracture energy, and air void content, while failure displacement was the least sensitive. Correlation analysis indicated that air void content was generally negatively correlated with the major crack-resistance indices, whereas flexural tensile strength was significantly positively correlated with pre-peak fracture energy. Overall, flexural tensile strength and pre-peak fracture energy can serve as useful key indicators for evaluating the evolution of crack resistance of asphalt concrete under coupled UV aging and freeze-thaw cycles within the scope of this study.

## Data Availability

All data, models, or code that support the findings of this study are available from the corresponding author upon reasonable request.
